# Supplementary material for: Expressing uncertainty in Human-Robot interaction
Source: PLoS One. 2020 Jul 16;15(7):e0235361. doi: 10.1371/journal.pone.0235361 (PMC7365469; doi:10.1371/journal.pone.0235361)
Supplement: Attachment — Submitted filename: response-reviews.pdf [file pone.0235361.s001.pdf]

Response to reviews

Christoph Bartneck, Elena Moltchanova

26 March 2020

1 Reviewer 1

One major concern from me is that the conclusion is very weak. Through the user studies, there seems no clear conclusion on which way is better in expressing uncertainties.

This is not a weak conclusion at all. Our study does indeed show that there is little difference between numerical expressions and verbal expressions. This is a surprising and interesting result. Science is not the hunt for statistically significant differences, but an attempt to learn. A negative result is still a result, and the dangers of reporting only (positive) statistically significant results are now becoming widely known. PLOS One is one of the leading journals that clearly declares that all papers that make a valuable contribution to the scientific literature, that are replicable, that are clearly written, and whose conclusions are supported by the data deserve publication (https://doi.org/10.1371/journal.pbio.0040401).

The decision trees that the authors proposed did not outperform the naive method. Hence, I think that the authors should try to explore more aspects of the work and make it contribute more to the community.

This comment is unhelpfully vague. The naive method and the trees are both statistical modelling methods, which make certain assumptions, and are appropriate for the data set in question. We thus applied both. The fact that the two models perform equally well does not mean that further modelling is needed. Perhaps, if the reviewer would like to be more explicit about what (s)he means by "exploring more aspects of the work and make it contribute more to the community".

Some minor comments focus on the writing in the conclusion part. Some of the expressions are very confusing to read. For instance, I cannot interpret the exact meaning of "we can predict the percentage response exactly around 50% of the time and within 10% of the true response 80% of the time".

We agree, that the sentences make for slow reading, but we are not sure what causes confusion. The first part means what it says. We can use our model to predict what percentage probability the respondent will assign to a certain verbal prompt. Our "guess" will be exactly right half the time and will be within 10% of the actual response 80% of the time.

The authors should summarize their results and findings in a more clear way.

This is a very general suggestion and it would be utmost helpful if the reviewer could please let us know what he/she thinks to be unclear. We could then focus our attention on those issues.

2 Reviewer 2

Make sure it is clear when presenting percentages that the meaning of the measurement. For example, a very brief explanation of what the apparent error rate is measuring could help understanding.

We define the apparent error rate in line 153 as the proportion of incorrect predictions. This is a standard textbook definition, and we are not sure whether it can be said any clearer.

Discuss how the decision tree is constructed in more detail.

In line 132 we explicitly refer to the textbook by Friedman, Hastie, and Tibshirani, which explains how the trees are constructed. We feel that such a textbook tutorial is necessary to fully understand the method and it goes beyond the scope of this paper to explain it. An interested reader can also simply use the existing function in the statistical software R, which has exactly the same format as the function for fitting a linear model. Whereas anyone interested in theory or algorithmic implementation can refer to the above textbook or to many of tutorials available on the internet.

More discussion of the low p-values (line 304) would be useful. I would make it more clear why more emphasis is put on the other statistical measures instead of the p-value. You could also discuss more about the percentage values in Fig 9 and 10.

In line 304 we do not speak of p-values, but rather importance scores, which we introduce in lines 155-165. We do understand, however, that the readership used to seeing p-values might automatically interpret them as such. We have therefore changed the text in lines 301-316 and the table legend to emphasize that we are talking about importance scores rather than p-values. We have also added another reference to Lin 2013, which explains why p-values are not useful for large data sets.

Minor comments: In line 190, there may be a typo in the prompt as it is not grammatically correct. I'd recommend including the abbreviation (AER) after "apparent error rate" at the beginning of sentence in line 284 for ease of reading.

We added the AER acronym as instructed.

The sentence on line 362 which starts "There appears to be more points..." is a strange phrase to use since it could be quantitatively determined whether or not the upper quartile of Fig 4 has more points than Fig 2.

Changed to "There are more points..."

3 Reviewer 3

The underlying data is not available to the reviewer in the time of the review, e.g. through a provisional DOI or a private reviewer URL link. Thus, point 3 on data availability is answered as No. The authors in their submission state that "they will publish the underlying data with the Open Science Foundation (https://osf.io/) upon the acceptance of our manuscript."

We will indeed make our data available at the Open Science Foundation upon acceptance of our manuscript. If they reviewers prefer, then we could make the data available through a private message.

Major comments:

The link on how does the study relate to previous work could be made clearer. In particular, are there other work that follow on the reference 30, that which the phrases used to build uncertainty expressions are taken from?

The study referenced in 30 was amongst the first in the field and was not published in the traditional academic way. Hence it did not attract as much attention as it should (46 citations since 1977). We carefully worked through the those citations and included those relevant to our study. We added a discussion about how the phrases used in 30 were used in the literature and how our results compare to them. In the introduction we offer a selection of more recent and relevant studies that tried to associate numerical expression of uncertainty to verbal expression of uncertainty. We already discussed our results within the context of those studies.

Amazon Mechanical Turk (MTurk) is used to sample the general population. In addition to seeing whether sex and age impact the interpretations, it could also be relevant to check their familiarity with probability theory, e.g. fundamentals like probabilities summing to one, or their background, profession or education.

There are many variables that could be considered in future experiments. We did record the basic demographic information. We added a discussion and references to dedicated studies that investigated the characteristics of the MTurk workers, including their occupation, psychological profile and ethnicity.

Minor comments:

- lines 94-95, the example expression seems to present high certainty for the event that a vehicle "avoids" collision is unlikely to occur. Is this the intended meaning, i.e. is it rather meant for the collision itself as an event being unlikely?

We modified the sentence to make it clear what event we are talking about.

- Fig 1 could be replaced with the high resolution original, and also credit the original work of license CC-BY and the two authors. at https://ourworldindata.org/uploads/2019/05/Cause of-death-in-USA-vs.-media-coverage.png

We modified the original figure by removing the small print below the graph that would be unreadable at this scale. We credited the work not only to its image, but also to its article. We added the the specific Creative Commons license to the caption.

- for naming consistency, it could be better to refer to the "uncertainty" prompt always as uncertainty (rather than in some places "certainty").

We changed all the prompt labels to "uncertainty".

- for naming consistency, Fig. 9 and Fig. 10 could use the same indices while referring to the agents (0, 1 vs. 1, 2).

We have realised that the Agent does not appear in the decision tree in Figure 9 (as the variable does not have a strong enough effect on choice). We have therefore corrected the legend in Figure 9 to read 1=you, 2= autonomous vehicle.

- line 56, typo: rendering "¡"0.01

Fixed.

- line 59, typo : Previous work ed

Fixed.

- line 65, could add AMT as an alternative abbreviation i.e. Amazon Mechanical Turn (AMT, MTurk)

Fixed.

- lines 73-74: the work is by Robert Sparrow and Mark Howard

Fixed.

- lines 47-48, should reference the Wittgenstein's quote from Tractatus.

Fixed.

- line 289 and caption of Fig. 8: "Figure" 6.

Fixed.

- line 364 - show"n"

Fixed.

- in the references , few punctuation-related issues e.g. ";." in 16 and 18, missing punctuation etc.

fixed
